# Phytochemical Analysis by HPLC–HRESI-MS and Anti-Inflammatory Activity of *Tabernaemontana catharinensis*

**DOI:** 10.3390/ijms19020636

**Published:** 2018-02-24

**Authors:** José Ivan Marques, Jovelina Samara Ferreira Alves, Manoela Torres-Rêgo, Allanny Alves Furtado, Emerson Michell da Silva Siqueira, Eder Galinari, Daline Fernandes de Souza Araújo, Gerlane Coelho Bernardo Guerra, Eduardo Pereira de Azevedo, Matheus de Freitas Fernandes-Pedrosa, Silvana Maria Zucolotto

**Affiliations:** 1 Laboratory of Pharmacognosy (PNBio), Department of Pharmaceutical Sciences, Faculty of Pharmacy, Federal University of Rio Grande do Norte, Avenida General Gustavo Cordeiro de Farias, S/N, Petrópolis 59012-570, Natal, Brazil; jsfa.farma@gmail.com (J.S.F.A.); siqueira_emerson@hotmail.com (E.M.d.S.S.); edergalinari@yahoo.com.br (E.G.); 2 Laboratory of Technology and Pharmaceutical Biotechnology (Tecbiofar), Department of Pharmaceutical Sciences, Faculty of Pharmacy, Federal University of Rio Grande do Norte, Avenida General Gustavo Cordeiro de Farias, S/N, Petrópolis 59012-570, Natal, Brazil; manoelatorres_rn@hotmail.com (M.T.-R.); allannyfurtado@hotmail.com (A.A.F.); mpedrosa@ufrnet.br (M.d.F.F.-P.); 3 Faculty of Health Sciences of Trairí, Federal University of Rio Grande do Norte, Avenida Trairí, S/N, Centro 59200-000, Santa Cruz, Brazil; daline_araujo@yahoo.com.br; 4 Department of Biophysics and Pharmacology, Bioscience Center, Campus Universitário, Federal University of Rio Grande do Norte, Avenida Senador Salgado Filho, 3000, Lagoa Nova 59072-970, Natal, Brazil; gerlaneguerra@hotmail.com; 5 Graduate Program of Biotechnology, Laureate Universities, Universidade Potiguar (UnP), Avenida Senador Salgado Filho, 1610, Lagoa Nova 59056-000, Natal, Brazil; eduardo.azevedo@unp.br

**Keywords:** Apocynaceae, flavonoids, herbal drug, carrageenan, zymosan

## Abstract

*Tabernaemontana catharinensis* (Apocynaceae) has been popularly used by folk medicine because of its anti-inflammatory, analgesic, and antiophidic properties. This study aims to analyze the flavonoids composition of the hydroethanolic extract and of the ethyl acetate (EtOAc) and butanol (BuOH) fractions of *T. catharinensis* leaves, as well as to evaluate their anti-inflammatory activity using in vivo models. The phytochemical profile, determined by High-Performance Liquid Chromatography–High-Resolution Electrospray Ionization-Mass Spectrometry (HPLC–HRESI-MS), showed the presence of flavonoids mainly having an isorhamnetin nucleus. The anti-inflammatory activity was evaluated in carrageenan-induced paw edema (pre- and post-treatment) with oral administration of a *T. catharinensis* hydroethanolic extract (50, 100, and 150 mg/kg) and of organic fractions (50 mg/kg). The extract and fractions showed antiedematogenic activity by decreasing myeloperoxidase (MPO) production. In the zymosan-air-pouch model, the extract and fractions inhibited leukocyte migration and significantly decreased the levels of various proteins, such as MPO, interleukin (IL)-1β, and tumor necrosis factor (TNF)-α. The cytotoxicity was evaluated by the 3-(4,5-dimethylthiazol-2-yl)-2,5-diphenyltetrazolium bromide (MTT) assay, which revealed no cytotoxicity of the extract and the fractions. These results suggest that the hydroethanolic extract and organic fractions of *T. catharinensis* leaves have sufficient anti-inflammatory activity to support the popular use of this plant in the treatment of inflammatory disorders.

## 1. Introduction

Inflammation is a complex biological process that involves several vascular and cellular mechanisms. The acute phase of the inflammatory process is characterized by increased vascular permeability, resulting in the accumulation of a neutrophil-rich fluid and pro-inflammatory mediators, such as histamine, serotonin, bradykinin, and prostaglandins [1,2,3,4]. The treatment of inflammatory disorders usually consists of administering nonsteroidal anti-inflammatory drugs (NSAIDs) and, in specific cases, anti-inflammatory steroids. However, the chronic use of steroids and NSAIDs is associated with severe side effects, such as gastric or intestinal ulceration, kidney disorders, edema, sodium retention, and arterial hypertension [5,6].

It was reported elsewhere that the potential anti-inflammatory drugs discovered in the last decade are mostly originated from natural sources [7,8]. In fact, several anti-inflammatory drugs were derived from the secondary metabolites of phenolic compounds, such as flavonoids [9]. The anti-inflammatory mechanism of action of phenolic compounds and flavonoids involves the inhibition of inflammatory mediators, such as inducible nitric oxide synthase (iNOS), cyclooxygenase (COX-2), and the cytokines IL-1β, TNF-α, and IL-10 [10]. 

Among the plants that have been popularly used by folk medicine practitioners, *T. catharinensis* (Apocynaceae) has shown anti-inflammatory activity [11]. In addition, antiophidic, analgesic and vermifuge are some of the properties that have been attributed to this plant. To the best of our knowledge, only two studies investigated the anti-inflammatory activity of *T. catharinensis* [12,13]. In one study, the alkaloid-free aqueous (100, 150, and 200 mg/kg) and ethanolic extracts (75 and 150 mg/kg) of *T. catharinensis* leaves were investigated in the carrageenan-induced paw edema mo­del after oral administration, but the extracts were ineffective in reducing edema. The extracts were active only when administered by intraperitoneal (i.p.) route [12,13]. In another study, the ethanolic extract from the stem bark of *T. catharinensis*, administered as a pretreatment at the dose of 150 mg/kg, reduced the edema in the same model [13].

Regarding the chemical composition of the extracts of *T. catharinensis* leaves, the literature describes indole alkaloids as the main secondary metabolites [14,15,16,17,18,19]. Therefore, the phytochemical identification of *T. catharinensis* is very limited, and a more detailed characterization becomes necessary in order to identify other secondary metabolites that might be responsible for the anti-inflammatory activity of this plant.

Considering that only few reports have been published about the anti-inflammatory activity of *T. catharinensis* and that there are no studies associating the pharmacological effects with the phytochemical composition of the plant-derived products, this study aims to perform a more in-depth investigation about the anti-inflammatory properties of *T. catharinensis* using different in vivo models. In addition, this study aims to analyze the phytochemical profile of the hydroethanolic extract of *T. catharinensis* leaves as well as of its ethyl acetate and butanol fractions.

## 2. Results

### 2.1. Thin-Layer Chromatography (TLC) Profile of the Hydroethanolic Extract and Organic Fractions of T. catharinensis Leaves

Thin-layer chromatography (TLC) analysis of the hydroethanolic extract and fractions of *T. catharinensis* leaves showed a wide variety of flavonoids. The plate was initially developed with sulfuric vanillin regent, and yellow spots were visualized in the hydroethanolic extract as well as in the ethyl acetate (EtOAc) and butanol (BuOH) fractions. Next, each plate was sprayed with the NP reagent (1% diphenylboryloxyethylamine in methanol; Sigma-Aldrich^®^, St. Louis, MO, USA) and further submitted to UV light (365 nm) exposure that revealed yellow and blue spots, which suggested the presence of flavonoids and phenolic derivatives, respectively [20,21]. Since there are several reports in the literature describing the anti-inflammatory activity of flavonoids [22], and considering that our TLC analysis indicated the presence of flavonoids in the hydroethanolic extract of *T. catharinensis* leaves, we hypothesized that enriched flavonoid fractions could be obtained from the EtOAc and BuOH fractions. The TLC analysis of these fractions showed similar profiles when compared to that of the hydroethanolic extract. However, the EtOAc fraction presented yellow bands in the upper part of the plate, whereas the BuOH fraction showed yellow and blue bands, suggesting the presence of flavonoids and phenolic derivatives, respectively.

### 2.2. High-Performance Liquid Chromatography–High-Resolution Electrospray Ionization-Mass Spectrometry (HPLC–HRESI-MS) Analysis of the Hydroethanolic Extract of T. catharinensis Leaves

**Figure 1:**
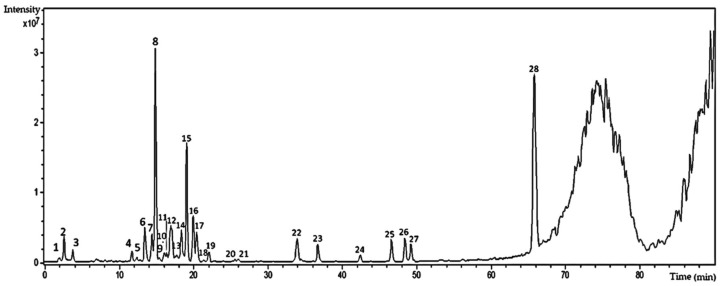
Chromatogram of the chemical profile of the hydroethanolic extract of *T. catharinensis* leaves obtained by High-Performance Liquid Chromatography (HPLC, Shimadzu LC-20AD, Shimadzu, Duisburg, Germany) with a photodiode detector (DAD, Shimadzu SPD-M20A) coupled with a mass spectrometer (micrOTOF II, Bruker Daltonics, Billerica, MA, USA) equipped with an electrospray ionization (ESI) source and a time-of-flight (TOF) type analyzer in negative mode. Experimental conditions: capillary voltage 3.2 kV, desolvation temperature 300 °C, gas flow 4 L∙min^−1^, and gas pressure 0.4 Bar. The spectra were analyzed using the Data Analysis software Bruker Daltonics. Nitrogen was used as a drying and nebulizing gas.

The HPLC–HRESI-MS analysis of the hydroethanolic extract of *T. catharinensis* leaves obtained in the negative ionization mode allowed the visualization of 28 peaks (Figure 1), 10 of which could be identified using mass spectra and UV data obtained from Massbank databases (available at http://www.massbank.jp) and from previous reports found elsewhere.

The majority of the peaks observed in Figure 1 is characteristic of the UV spectra of flavonoids, with two absorption bands between 254 and 265 nm, corresponding to the absorption of the benzoyl group of ring A, and bands between 346 and 354 nm, corresponding to the cinnamyl group and related to rings B and C [20,21]. These results corroborate the previous TLC finding that flavonoids are present in the hydroethanolic extract of *T. catharinensis* leaves. In addition, through HPLC–ESI-MS (negative mode) analysis, some flavonoids present in the extract of *T. catharinensis* leaves were identified, and most of them presented the isorhamnetin nucleus in their structure (Table 1).

### 2.3. Cytotoxicity Assay

**Figure 2:**
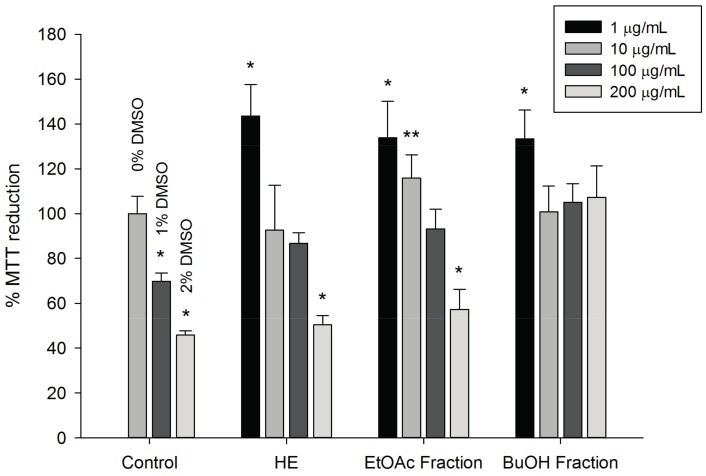
The MTT assay performed with a macrophage cell line (RAW 264.7). The BuOH fraction (1, 10, 100, and 200 µg/mL) was dissolved in distilled water resulting in a final concentration of 10 mg/mL, whereas the hydroethanolic extract—(HE, 1, 10, 100, and 200 µg/mL) and the EtOAc fraction (1, 10, 100, and 200 µg/mL) were dissolved in dimethyl sulfoxide (DMSO), resulting in a final concentration of 10 mg/mL. DMSO at two concentrations (1% and 2%) were used to demonstrate its effect on MTT concentration. ** p < 0.01 and * *p* < 0.05 and tested group compared to the control group (0% DMSO).

The Methyl tetrazolium (MTT) assay was used to evaluate the cytotoxicity of the hydroethanolic extract of *T. catharinensis* leaves and of the EtOAc and BuOH fractions at concentrations of 1, 10, 100, and 200 μg/mL, using a blood-derived murine macrophage cell line (RAW 264.7, Sigma-Aldrich^®^, St. Louis, MO, USA) during a 24 h test. The results (Figure 2) suggested that the extract and fractions of *T. catharinensis* did not show cytotoxicity in RAW 264.7 cells at the concentrations used in this study. In addition, the ability of the fractions and extract to reduce MTT was investigated by performing the experiment without the RAW 264.7 cell line, using the same experimental conditions, and no change in MTT was observed. Therefore, the cell viability assay showed that the hydroethanolic extract and the EtOAc and BuOH fractions of *T. catharinensis* leaves at concentrations from 1 to 200 μg/mL are not cytotoxic.

### 2.4. Anti-Inflammatory Effect of the Pretreatment with the Hydroethanolic Extract and the Fractions of T. catharinensis Leaves in the Carrageenan-Induced Paw Edema Model

**Figure 3:**
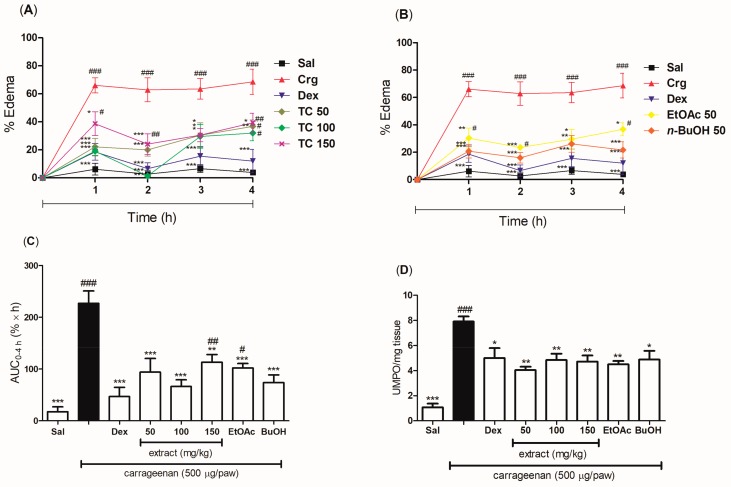
Evaluation of the anti-inflammatory effect of the pretreatment with the hydroethanolic extract and the ethyl acetate (EtOAc) and *n*-butanol (BuOH) fractions of *T. catharinensis* leaves in the carrageenan-induced paw edema model. BALB/c mice were treated orally with saline, the hydroethanolic extract at doses of 50, 100, or 150 mg/kg, the EtOAc and BuOH fractions at a dose of 50 mg/kg, and dexamethasone at 2 mg/kg. The edema was measured at different time points (1, 2, 3, and 4 h) after the administration of *λ*-carrageenan (500 μg/paw). After 4 h, the paw was removed and the enzymatic activity was measured. One unit of myeloperoxidase (MPO) activity (UMPO) was defined as the degradation of one micromole of peroxide per minute. (**A**) Percentage of edema (0–4 h) in mice pretreated with the hydroethanolic extract; (**B**) Percentage of edema (0–4 h) in mice pretreated with the EtOAc and BuOH fractions; (**C**) Area under the curve (AUC) for the percentage of paw edema as a function of time (0–4 h) in mice pretreated with the hydroethanolic extract and fractions; (**D**) MPO activity in paw tissues of mice pretreated with the hydroethanolic extract and fractions. ^###^ *p* < 0.001, ^##^ *p* < 0.01, and ^#^ *p* < 0.05 compared with the group treated with saline; *** *p* < 0.001, ** *p* < 0.01 and * *p* < 0.05 compared with the carrageenan group. Sal: saline (0.9 mg/mL); Crg: carrageenan (500 µg/paw); Dex: dexamethasone (2.0 mg/kg); TC 50: *T. catharinensis* extract (50 mg/kg); TC 100: *T. catharinensis* extract (100 mg/kg); TC 150: *T. catharinensis* extract (150 mg/kg); EtOAc 50: ethyl acetate fraction (50 mg/kg); BuOH 50: 𝑛-butanol fraction (50 mg/kg).

The acute anti-inflammatory effect of an oral pretreatment with the hydroethanolic extract and the EtOAc and BuOH fractions of *T. catharinensis* leaves was evaluated in the carrageenan-induced paw edema model. The mice were treated orally with saline and, thirty minutes later, with an intraplantar injection of carrageenan, whereby they exhibited a severe edema formation at all time points (1, 2, 3, and 4 h), as well as an increased in myeloperoxidase levels after 4 h of edema induction. All groups treated orally with the hydroethanolic extract at doses of 50, 100, and 150 mg/kg showed a significant inhibition of edema formation during 4 h of experiment when compared to the groups treated with either the negative control (saline group) or the positive control (carrageenan group) (Figure 3A). Similarly, the EtOAc and BuOH fractions at the dose of 50 mg/kg significantly reduced the edema (Figure 3B). Considering the average of the Area Under the Curve (AUC) 0–4 h, the maximum antiedematogenic effect seemed to be achieved at doses of 50 and 100 mg/kg of extract, with a reduction of 59% and 71% of the edema, respectively. The EtOAc and BuOH fractions were able to reduce the edema of 55% and 67%, respectively (Figure 3C). The different doses of the extract, as well as the EtOAc and BuOH fractions (50 mg/kg), significantly inhibited the production of myeloperoxidase (Figure 3D). In addition, the reduction of the edema in the group treated with dexamethasone was very similar to that observed in mice treated with the hydroethanolic extract or the fractions of *T. catharinensis* leaves, whereas the decrease in myeloperoxidase levels was greater in the groups treated with the extract and the EtOAc fraction than in the group treated with dexamethasone. 

### 2.5. Anti-Inflammatory Effect of the Post-Treatment with the Hydroethanolic Extract and the Fractions of T. catharinensis Leaves in the Carrageenan-Induced Paw Edema Model

**Figure 4:**
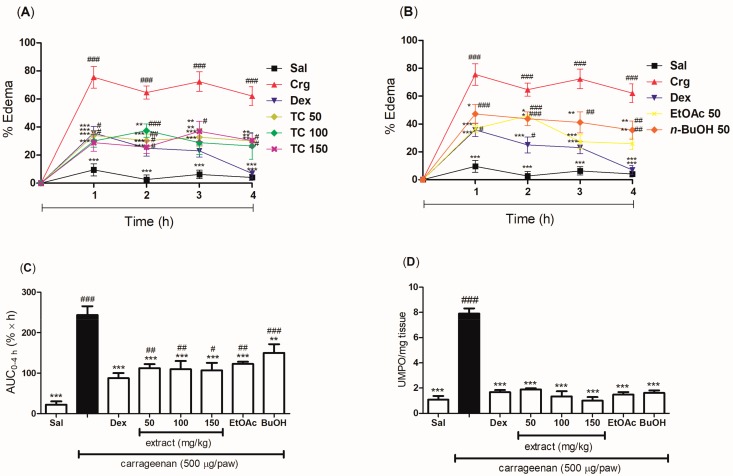
Evaluation of the anti-inflammatory effect of the post-treatment with the hydroethanolic extract and the ethyl acetate (EtOAc) and *n*-butanol (BuOH) fractions of *T. catharinensis* leaves in the carrageenan-induced paw edema model. BALB/c mice were treated orally with saline, the hydroethanolic extract at doses of 50, 100, or 150 mg/kg, the ethyl acetate (EtOAc) and *n*-butanol (BuOH) fractions at the dose of 50 mg/kg, and dexamethasone (2 mg/kg). The edema was measured at different time points (1, 2, 3, and 4 h) after the administration of *λ*-carrageenan (500 μg/paw). After 4 h, the paw was removed and the enzymatic activity was measured. One unit of MPO activity (UMPO) was defined as one micromole of peroxide degraded per minute. (**A**) Percentage of edema (0–4 h) in mice post-treated with the hydroethanolic extract; (**B**) Percentage of edema (0–4 h) in mice post-treated with the EtOAc and BuOH fractions; (**C**) AUC of the percentage of edema as a function of time in hours (0–4 h) in mice post-treated with the hydroethanolic extract and the fractions; (**D**) MPO activity in the paws of mice post-treated with the hydroethanolic extract and the fractions. ^###^ *p* < 0.001, ^##^ *p* < 0.01 and ^#^ *p* < 0.05 compared with the group treated with saline; *** *p* < 0.001, ** *p* < 0.01, and * *p* < 0.05 compared with the carrageenan group. Sal: saline (0.9 mg/mL); Crg: carrageenan (500 µg/paw); Dex: dexamethasone (2.0 mg/kg); TC 50: *T. catharinensis* extract (50 mg/kg); TC 100: *T. catharinensis* extract (100 mg/kg); TC 150: *T. catharinensis* extract (150 mg/kg); EtOAc 50: ethyl acetate fraction (50 mg/kg); BuOH 50: 𝑛-butanol fraction (50 mg/kg).

In order to evaluate the post-treatment effect of *T. catharinensis*, its hydroethanolic extract and fractions were administered orally to mice, and their anti-inflammatory effects were investigated using the carrageenan-induced paw edema model. The groups treated orally with doses of 50, 100, and 150 mg/kg of the hydroethanolic extract showed a marked reduction in the edema within a period of 4 h (Figure 4A). Similarly, the EtOAc and BuOH fractions at the dose of 50 mg/kg showed a significant edema reduction (Figure 4B). Taking into account the progress of the edema reduction within the course of 4 h (AUC_0–4 h_), a similar antiedematogenic effect could be observed among the different doses of the hydroethanolic extract used in this study (50, 100, and 150 mg/kg), indicating that they were able to reduce the edema of 54%, 55%, and 56%, respectively. The EtOAc and BuOH fractions showed edema inhibition of 50% and 38%, respectively (Figure 4C). Regarding the myeloperoxidase assay, the hydroethanolic extract and fractions showed similar results as dexamethasone (Figure 4D). It is worth pointing out that in all groups the post-treatment was more effective in decreasing the levels of myeloperoxidase than the pretreatment.

### 2.6. Evaluation of the Anti-Inflammatory Effect of the Hydroethanolic Extract and the Fractions of T. catharinensis Leaves in the Zymosan-Induced Air-Pouch Model

**Figure 5:**
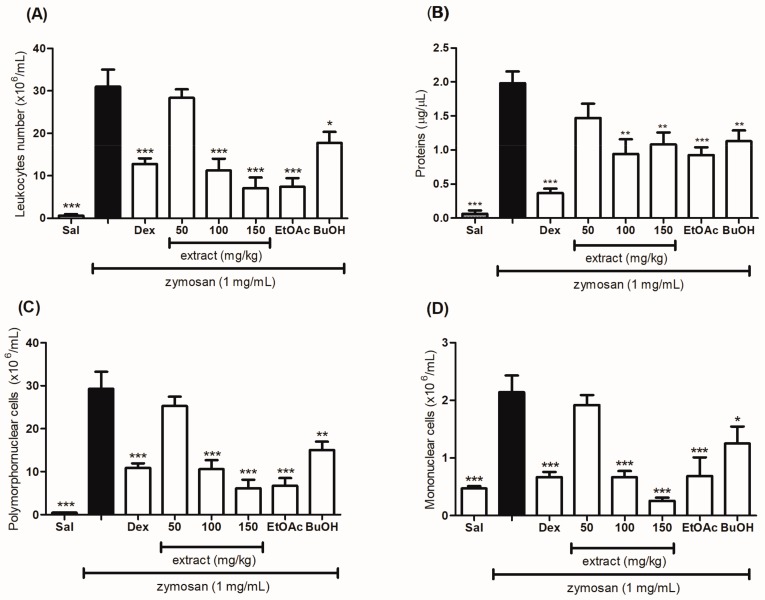
Evaluation of the anti-inflammatory effect of the hydroethanolic extract and the ethyl acetate (EtOAc) and *n*-butanol (BuOH) fractions of *T. catharinensis* leaves on leukocytes migration and protein production in the zymosan-induced air pouch model. Swiss mice were treated orally with saline solution, the hydroethanolic extract at doses of 50, 100, or 150 mg/kg, the ethyl acetate (EtOAc) and *n*-butanol (BuOH) fractions at the dose of 50 mg/kg, and dexamethasone (2.0 mg/kg). After thirty minutes, a zymosan solution (1 mg/mL) or saline (1 mL) was injected into the animals’ air pouch. After six hours, the exudate was collected with 2 mL of saline and centrifuged, and both total and subpopulations counts of leukocytes were determined. The total leucocytes count was carried out using a *Neubauer* chamber (**A**). The cell subpopulations count, polymorphonuclear (**C**) and mononuclear (**D**), was determined by counting 100 cells using a hemocytometer. The supernatants were collected for the determination of total proteins by Bradford’s assay (**B**). Each column represents the mean of the values obtained from five animals, and the vertical lines indicate the standard errors of the mean (SEM). *** *p* < 0.001, ** *p* < 0.01 and * *p* < 0.05 compared with the zymosan group (black bar). Sal: saline (0.9 mg/mL); Dex: dexamethasone (2 mg/kg); TC 50: *T. catharinensis* extract (50 mg/kg); TC 100: *T. catharinensis* extract (100 mg/kg); TC 150: *T. catharinensis* extract (150 mg/kg); EtOAc: ethyl acetate fraction (50 mg/kg); BuOH: *n*-butanol fractions (50 mg/kg).

Severe inflammation is induced in the zymosan-induced air pouch model, whose response is characterized by intense cell migration, plasma exudation, and increase in total proteins towards the animal’s cavity. The oral treatment with the extract (50, 100, and 150 mg/kg) and the EtOAc and BuOH fractions (50 mg/kg) of the *T. catharinesis* leaves was effective in decreasing cell migration into the animals’ air pouch when the treated mice were compared to the group that received only saline (orally) or zymosan (subcutaneously), as shown in Figure 5A. The hydroethanolic extract at the dose of 150 mg/kg and the EtOAc fraction inhibited cell migration to almost 80% (Table 2).

For the polymorphonuclear (PMN) and mononuclear (MN) cells (Figure 5C,D, respectively), migration inhibition was observed for both the hydroethanolic extract and the fractions. For the quantification of total proteins, a decrease was observed in the groups treated with the extract and the fractions (Figure 5B). In addition, these groups showed similar results as those of the group treated with dexamethasone.

### 2.7. Evaluation of the Effect of the Hydroethanolic Extract and the Fractions of T. catharinensis Leaves on the Levels of Myeloperoxidase and Cytokines

**Figure 6:**
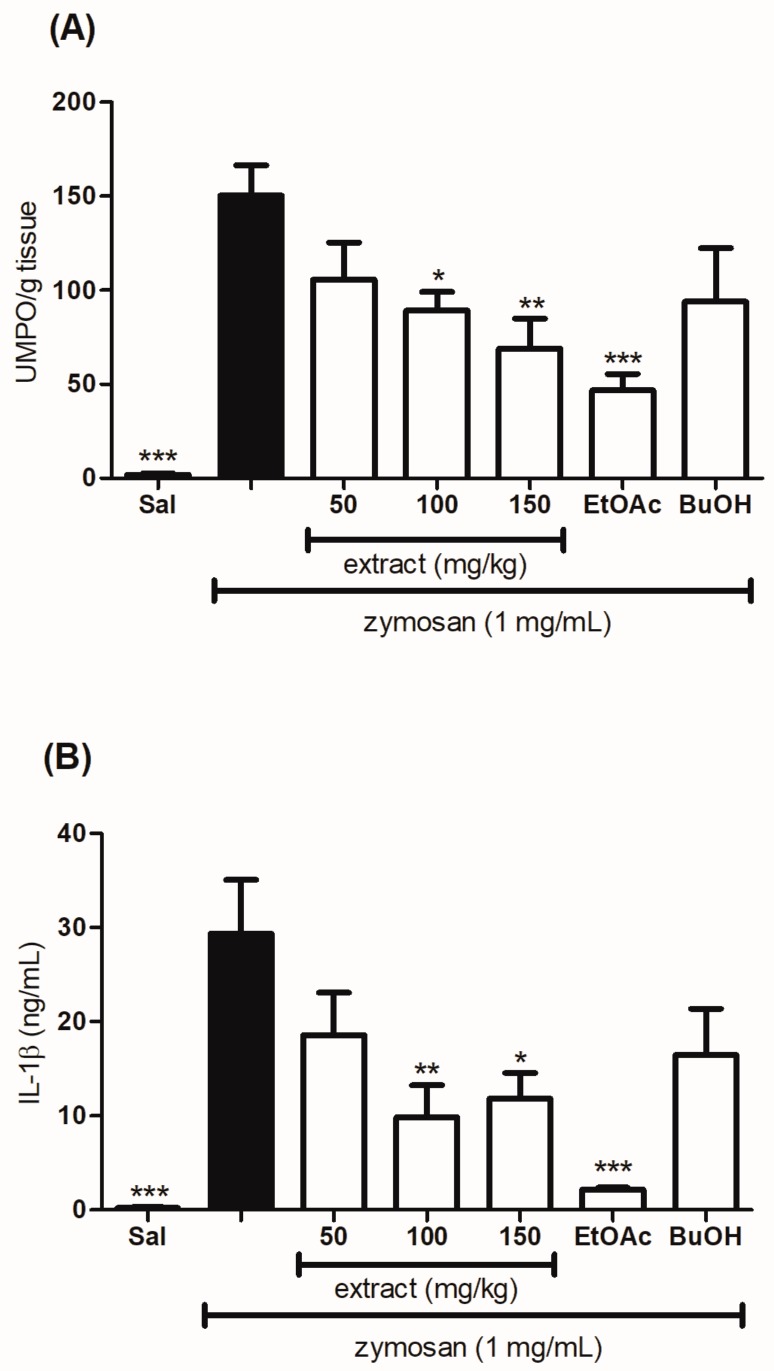

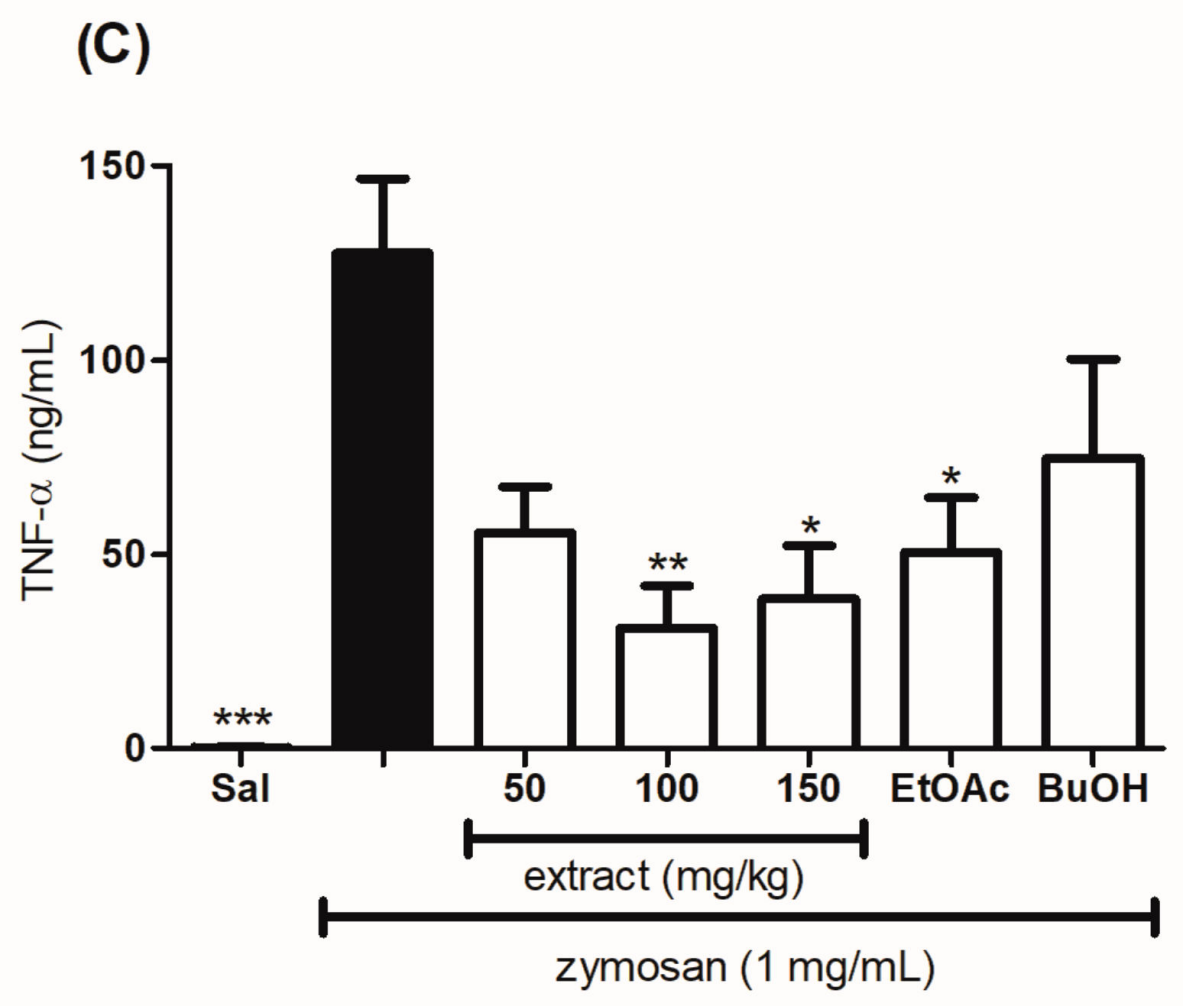
Evaluation of the anti-inflammatory effect of the hydroethanolic extract and the fractions of *T. catharinensis* leaves on the levels of myeloperoxidase and cytokines in the zymosan-induced air pouch model. Swiss mice were treated orally with a saline solution, the hydroethanolic extract at doses of 50, 100, and 150 mg/kg, the ethyl acetate (EtOAc) and *n-*butanol (BuOH) fractions at the dose of 50 mg/kg, and dexamethasone (2.0 mg/kg). After thirty minutes, a zymosan solution (1 mg/mL) or saline (1 mL) was injected into the animals’ air pouch. After six hours, the exudate was collected with 2 mL of saline, and the levels of myeloperoxidase (**A**), cytokines interleukin (IL)-1β (**B**), and tumor necrosis factor TNF-α (**C**) were determined. Each column represents the mean of the values obtained from five animals, and the vertical lines indicate the standard errors of the mean (SEM). *** *p* < 0.001, ** *p* < 0.01, and * *p* < 0.05 compared with the zymosan group (black bar). Sal: saline (0.9 mg/mL); *T. catharinensis* extract (50 mg/kg); TC 100: *T. catharinensis* extract (100 mg/kg); TC 150: *T. catharinensis* extract (150 mg/kg); EtOAc: ethyl acetate fraction (50 mg/kg); BuOH: *n*-butanol fractions (50 mg/kg).

As expected, the animals treated with a subcutaneous administration of zymosan increased the production of IL-1β, TNF-α, and myeloperoxidase. On the other hand, the animals treated orally with different doses of the extract (100 and 150 mg/kg), as well as with the EtOAc and BuOH fractions of *T. catharinensis* leaves at the dose of 50 mg/kg showed a significant reduction in the levels of myeloperoxidase (Figure 6A) when compared to the groups treated with either the negative control (saline group) or the positive control (carrageenan group). In addition, the hydroethanolic extract and the fractions were able to decrease the production of cytokines IL-1β (Figure 6B) and TNF-α (Figure 6C).

## 3. Discussion

Anti-inflammatory drugs usually act by either antagonizing or inhibiting specific enzymes involved in the inflammatory process. Nonsteroidal anti-inflammatory drugs (NSAIDs) inhibit cyclooxygenase (COX), whereas steroidal anti-inflammatory drugs (SAIDs) block the mediators of the inflammatory response [23,24,25]. Although SAIDs and most NSAIDs are effective to manage inflammation, their long-term use is associated with severe side effects [26,27]. In this context, the plant biomass has emerged as an alternative source for new bioactive substances as well as for the development of herbal medicines, which can be as effective as the synthetic drugs available in the market, but with fewer side effects [28]. 

Among the plants with a potential pharmacological application, *T. catharinensis* has been widely used by folk medicine practitioners for the treatment of some inflammatory diseases [12]. Despite its widespread use, only two reports have been published about the anti-inflammatory activity of *T. catharinensis*. Rates et al. (1993) investigated the anti-inflammatory activity of the aqueous and ethanolic extracts of *T. catharinensis* leaves using the carrageenan-induced paw edema model [13]. On the other hand, Gomes et al. (2009) evaluated the anti-inflammatory activity of the oral administration of bark extracts of *T. catharinensis* using the same experimental model [12]. Although *T. catharinensis* anti-inflammatory activity has been previously investigated [12,13], to the best of our knowledge, no study has reported an in-depth investigation of the anti-inflammatory activity of *T. catharinensis* leaves and a chemical characterization of their extract [29,30].

Therefore, the purpose of this study was to determine the phytochemical constituents of a hydroethanolic extract and of two enriched flavonoid fractions of *T. catharinensis* leaves, as well as to investigate their anti-inflammatory activity in more detail through the carrageenan-induced paw edema and the zymosan-induced air-pouch models. In addition, the anti-inflammatory activity was assessed by determining the levels of myeloperoxidase, proteins, and cytokines.

The hydroethanolic extract as well as the EtOAc and BuOH fractions of *T. catharinensis* leaves were characterized by HPLC–HRESI-MS, which allowed the identification of the main peaks attributed to the glycosylated flavonoids kaempferol, quercetin, and isorhamnetin. The peak with retention time of 15 min (Figure 1) was identified as isorhamnetin, in addition to the other glycosylated flavonoids quercetin and kaempferol (Table 1). This is the first time that such compounds have been identified in *T. catharinensis*, since the previous reports dealt only with the isolation and purification of alkaloids and their secondary metabolites from the *Tabernaemonta* genus [14,15,17]. In addition, only few reports are available on the characterization of phenolic compounds, because only their total quantification was performed without any identification of the individual compounds [31].

In this current study, the anti-inflammatory activity of the hydroethanolic extract and fractions of *T. catharinensis* leaves was evaluated using two in vivo models: the carrageenan-induced paw edema and the zymosan-induced air pouch. The former is the most commonly used model for evaluating the anti-inflammatory activity of plant extracts and herbal products [32,33] and it implies the use of carrageenan as the phlogistic agent. Carrageenan induces an acute inflammatory process associated with hyperalgesia and the release of inflammatory mediators, which is characteristic of an edematogenic response [34].

Three events are associated with the carrageenan-induced paw edema model. The first event, which occurs within the first 90 min, is characterized by vasodilation due to the release of serotonin and histamine. Between 90–150 min, a release of kinins occurs, which are responsible for the biosynthesis of prostaglandins. The last event, which occurs after 150 min, is characterized by an intensifying production of prostaglandins and a massive infiltration of polymorphonuclear leukocytes [32,33].

On the other hand, the air pouch model uses the inflammatory agent zymosan (glucan), which is a yeast cell wall extract. Its mechanism of action involves the activation of the inflammatory process via the complement system, inducing the secretion of lysosomal enzymes and the release of prostaglandins and leukotrienes [35].

In this study, the hydroethanolic extract as well as the EtOAc and BuOH fractions of *T. catharinensis* leaves significantly reduced the edema induced by carrageenan in mice, and the hydroethanolic extract showed effectiveness in both the pre- and the post-treatment, especially at the pretreatment dose of 100 mg/kg, which inhibited 97.8% of edema formation in 2 h. The BuOH fraction (50 mg/kg) produced a similar response (74.6%). It is worth to point out that dexamethasone, which was used as the standard anti-inflammatory agent, showed a percentage of edema reduction of 89.9%, being less effective than the hydroethanolic extract of *T. catharinensis* leaves.

The post-treatment with the hydroethanolic extract and the fractions also inhibited edema formation but to a lesser extent when compared with the pretreatment. The post-treatment with the hydroethanolic extract inhibited 62% of the edema at the dose of 100 mg/kg. On the other hand, the EtOAc fraction was more effective than the BuOH one, with a percentage of edema inhibition of 62% at the dose of 50 mg/kg. The group treated with dexamethasone showed a percentage of edema inhibition of 88.8%.

In order to understand the anti-inflammatory effect of the extract and fractions of *T. catharinensis* leaves, a myeloperoxidase (MPO) assay was conducted, as the presence of MPO is associated with exudation and cell migration [36]. MPO is an enzyme released by cells at the beginning of the inflammatory process, and, therefore, its level has been used as a parameter to evaluate inflammation [37]. The decrease in the levels of MPO after treatment with the hydroethanolic extract and the fractions of *T. catarinensis* seemed to be due to their ability to reduce the migration of both PMN and NM cells [38].

A decrease in leukocyte migration, observed through the decrease in MPO levels and leukocyte count, may be related, at least in part, to the glycosylated flavonoids isorhamnetin, kaempferol, and quercetin, which are present in the hydroethanolic extract and in the organic fractions of *T. catarinensis* leaves, as shown in the HPLC–HRESI-MS analysis. According to previous reports, the anti-inflammatory activity of these glycosylated flavonoids is due to a reduction in the expression of the vascular cell adhesion molecule-1 (VCAM-1), the intracellular adhesion molecule-1 (ICAM-1), and e-selectin, through a mechanism associated with cellular migration [39,40].

In addition to the edematogenic activity and the reduction of leukocyte migration, the hydroethanolic extract and the organic fractions of *T. catharinensis* leaves were capable of inhibiting the production of proinflammatory cytokines, such as IL-1β and TFN-α. These cytokines are responsible for most of the inflammatory disorders [41]. IL-1β is related to pain, whereas TFN-α is associated with cell death, both being markedly increased in tissue inflammation [42]. The hydroethanolic extract at the dose of 100 mg/kg showed the best activity in inhibiting the production of these cytokines. It seems that this inhibitory activity is associated with the flavonoids present in the *T. catharinensis* leaves. The effect of decreasing the production of cytokines has already been reported for isorhamnetin [43,44], kaempferol [40,45], and quercetin [46]. Since these flavonoids are present in the hydroethanolic extract as well as in the fractions of *T. catharinensis* leaves, an inhibitory effect on cytokines production contributing to the observed anti-inflammatory activity of *T. catharinensis* cannot be ruled out.

To the best of our knowledge, only two studies have investigated the anti-inflammatory activity of *T. catharinensis* [12,13]. The first study, which was conducted by Rates et al. (1993) [12], investigated the oral pretreatment with an alkaloid-free aqueous extract at doses of 100, 150, and 200 mg/kg and with an ethanolic extract at doses of 75 and 150 mg/kg in the carrageenan-induced paw edema model. The results showed that the extracts had no effect on edema reduction, only when administered by i.p. route. On the other hand, Gomes et al. (2009) [12,13] studied the pretreatment with an ethanolic extract of *T. catharinensis* stem bark in the carrageenan-induced paw edema, and observed a reduction in the edema only at the dose of 150 mg/kg. However, this study was carried out with another part of the plant.

Our present study showed that the hydroethanolic extract as well as the EtOAc and BuOH fractions of the *T. catharinensis* leaves presented significant anti-inflammatory activity in both the carrageenan-induced paw edema and the zymosan-induced air-pouch models. This is the first study that reports the anti-inflammatory activity of the hydroethanolic extract and of the fractions of *T. catharinensis* leaves. Another relevant finding is that the extract and fractions were not cytotoxic at the concentrations used in this study, as shown by the MTT assay.

Thus, the results obtained in this study seem to indicate that the hydroethanolic extract and the organic fractions of *T. catharinensis* leaves show sufficient anti-inflammatory activity to justify the popular use of this plant to treat inflammatory disorders. This activity seems to be related, at least in part, to the presence of flavonoids as well as to the inhibitory effect on the synthesis and activities of different pro-inflammatory mediators, such as eicosanoids, cytokines, and adhesion molecules [22].

Although the topical route maximizes the local effect and minimizes systemic toxicity, the majority of topical anti-inflammatory formulations contain corticosteroids, whose chronic use has been associated with numerous side effects [47,48]. In this context, topical formulations containing the hydroethanolic extract of *T. catharinensis* leaves seem to be an innovative and safer alternative for the treatment of topical inflammatory disorders, however further studies are needed to prove the efficacy and safety of the extract.

## 4. Materials and Methods

### 4.1. Plant Material

*T. catharinensis* leaves were collected in Ibiapina City, Ceara State, Brazil at coordinates 3°96′57″ S, 40°91′98″ W, in December of 2015. The sample was identified by botanist Dr. Leandro de Melo Versieux and deposited in the Herbarium of the Federal University of Rio Grande do Norte with voucher specimen number 20587 (29 November 2016). The collection of the plant material was conducted under the Brazilian Authorization and Biodiversity Information System (SISBIO) with process number 34,017 (17 April 2013), and the research by authorization from the National System for Management of Genetic Heritage and Associated Traditional Knowledge (SISGEN) n^o^. AF3D173.

### 4.2. Extract Preparation

*T. catharinensis* leaves (300 g) were dried at room temperature for five days and comminuted using an industrial blender. The extract was prepared by maceration with ethanol:water at the volume ratio of 70:30 (*v*/*v*) for 5 days. The obtained hydroethanolic extract was filtered and concentrated in a rotary evaporator at 50 °C (Büchi-Model V-700, Altendorfer Str. 3, Essen, Germany). The concentrated extract was lyophilized, yielding 55.3 g of dried hydroethanolic extract. The lyophilized extract was analyzed by HPLC–HRESI-MS, after solubilizing 1 mg in 1.0 mL of the initial mobile phase and filtering it with a CHROMAFIL^®^ XTRA syringe filter in a PVDF polypropylene shell (15 mm in diameter and pore size of 0.45 μm). On the basis of the TLC results, we opted for the fractionation of the extract to obtain enriched flavonoid fractions through a liquid–liquid extraction using organic solvents with different polarities: hexane, chloroform, ethyl acetate (EtOAc), and *n*-butanol (BuOH), three times each. The fractions were concentrated in a rotary evaporator at a temperature below 50 °C.

### 4.3. Thin-Layer Chromatography (TLC) Profile of the Leaves Extract

Thin-layer chromatography (TLC) analysis was carried out using silica gel F_254_ 20 × 20 aluminum plates (Merck, Darmstadt, Germany), using ethyl acetate:acetone:acetic acid:water (6:2:1:1 *v/v*) as the mobile phase. After development, the plates were dried and observed under UV light (254 and 365 nm). The plates were sprayed with 1% diphenylboryloxyethylamine in methanol (Sigma-Aldrich^®^, St. Louis, MO, USA) and visualized under UV 365 nm. The parameters analyzed were the retention factor (*Rf*), color, and behavior of the spots, which were compared with chromatographic profiles of reference substances reported in the literature [21].

### 4.4. High-Performance Liquid Chromatography–High-Resolution Electrospray Ionization-Mass Spectrometry (HPLC–HRESI-MS) Profile of the Leaves Extract

The hydroethanolic extract of *T. catharinensis* leaves was analyzed by high-performance liquid chromatography (HPLC, Shimadzu LC-20AD, Duisburg, Germany) with a photodiode detector (DAD, Shimadzu, Duisburg, Germany) coupled to a high resolution mass spectrometer (micrOTOF II, Bruker Daltonics, Bellerica, Massachusetts, USA) fitted with an electrospray ionization source (ESI) and a time-of-flight (TOF) analyzer. The MS/MS data was obtained in a mass spectrometer equipped with an electrospray ionization source and an ion-trap analyzer. The mass spectrometer conditions were: capillary voltage 3.2 kV, desolvation temperature 300 °C, gas flow 4 L min^−1^, and gas pressure 0.4 Bar. Nitrogen and helium were used as drying and collision gases, respectively. The data was analyzed using the Data Analysis 4.2 software (Bruker Daltonics, Billerica, MA, USA).

### 4.5. MTT Assay

A murine macrophage cell line (RAW 264.7) was seeded (7 × 10^3^ cells/well) in a 96-well plate and cultured in Dulbecco’s modified Eagle’s medium (DMEM) with 10% fetal bovine serum (FBS). Cells were incubated in a humidified atmosphere, at 37 °C with 5% CO_2_ for 24 h. The medium was further replaced by a fresh aliquot of DMEM without FBS, followed by incubation for 24 h. Then, the medium was replaced by 100 µL/well of DMEM + 10% FBS + the sample (1, 10, 100, or 200 µg/mL), and the plate was incubated for 24 h under the aforementioned conditions. Next, the medium was replaced by DMEM without FBS, containing 1 mg/mL of 3-(4, 5-dimethylthiazol-2-yl)-2,5-diphenyltetrazolium bromide (MTT), and the plate was incubated for additional 4 h. Finally, the solution of each well was replaced by 100 µL of 95% ethyl alcohol to solubilize the formazan crystals. The BuOH fraction was dissolved in distilled water (10 mg/mL), and the negative control cells were cultivated with only DMEM + 10% FBS. On the other hand, the hydroethanolic extract and the AcOEt fraction were dissolved in DMSO (10 mg/mL), therefore, the two DMSO controls were used at concentrations of 1% or 2%. The absorbance was read in a microplate reader at 570 nm, and the results were expressed as percentage of MTT reduction, as determined using the following equation:

% MTT reduction = (absorbance of sample/absorbance of control) × 100

### 4.6. Animals

Male and female Swiss and BALB/c mice (25–35 g), 6–8 weeks of age, were housed in a light-controlled room (12/12 h light/dark cycle) at a temperature of 22 ± 2 °C. The animals were provided by the Animal Facility of the Health Sciences Center at Federal University of Rio Grande do Norte (UFRN), Brazil. Each group comprised five animals (*n* = 5). After the experiments, the animals were euthanized with an overdose of xylazine and ketamine (10–100 mg/kg) by intraperitoneal injection. The experimental protocol was approved by the Committee for Ethics in Animal Experimentation of UFRN under the Protocol n° 063/2015 (4 November 2015), in accordance with ethical principles in animal research adopted by the Brazilian Society of Animal Science and the National Brazilian Legislation n^o^. 11.794/08, 8 October 2008.

### 4.7. Carrageenan-Induced Paw Edema Model

The in vivo anti-inflammatory activity of the pre- and post-treatment with the hydroethanolic extract as well as the EtOAc and BuOH fractions were evaluated using the carrageenan-induced paw edema model in mice, as previously described, with few modifications [49]. The first protocol was carried out by orally administering to BALB/c mice 300 μL of saline, dexamethasone (2.0 mg/kg), hydroethanolic extract (50, 100 or 150 mg/kg), or EtOAc and BuOH fractions (50 mg/kg) of *T. catharinensis* leaves. Thirty minutes after the treatment, the animals received an intraplantar injection of 50 µL of 1% 𝜆-carrageenan (Sigma^®^ Aldrich) or saline solution. The second protocol was carried out by administering an intraplantar injection of 50 µL of 1% 𝜆-carrageenan or saline solution. Thirty minutes after inducing inflammation, the mice were treated as aforementioned. At the end of the experiment, two parameters were evaluated: the percentage of edema reduction and myeloperoxidase (MPO) levels (methodology described below). The paw edema was expressed in millimeters (mm) and was calculated as the percentage of edema. The area under the time-course curve (AUC_0–4 h_) was also determined using the trapezoidal rule.

### 4.8. Zymosan-Induced Air-Pouch Model

The anti-inflammatory activity was also evaluated using the zymosan-induced air pouch model with some modifications [50,51]. Briefly, Swiss mice received 5 mL of sterile air subcutaneously, injected into the back of the animals. After three days, 2.5 mL of sterile air was injected into the cavity. Six days after the initial air injection, the animals received a zymosan solution (1 mg/mL) into the air pouch. Simultaneously, the mice were treated orally with 300 μL of saline, dexamethasone (2 mg/kg), hydroethanolic extract (50, 100 or 150 mg/kg), or EtOAc and BuOH fractions (50 mg/kg) of *T. catharinensis* leaves. After six hours, the animals were euthanized, and the exudates were harvested from each air pouch by washing with 2 mL of saline solution. The total number of leukocytes was determined using a *Neubauer* chamber with the aid of a Nikon ECLIPSE E200^®^ (Minato, Tokyo, Japan) microscope at 40× magnification. The results were expressed as leukocytes number per mL. In addition, the differential cell count as well as the myeloperoxidase (MPO), interleukin-1β (IL-1β), and tumoral necrose factor-α (TNF-α) levels were determined.

### 4.9. Differential Cell Count in the Exudate

The cell pellet was diluted in 1 mL of saline solution, and the polymorphonuclear and mononuclear cell count was determined on the basis of the count of 100 cells, using a hemocytometer. The slides were stained using rapid panoptic staining (Laborclin, Paraná, Brazil) and further examined under light microscopy (Nikon ECLIPSE E200^®^, Minato, Tokyo, Japan), at 100× magnification. The absolute values were expressed as leukocyte counts per mL, as previously described [52,53].

### 4.10. Determination of Total Proteins

The supernatants were collected for the determination of total proteins using the Bradford’s assay. An amount of 10 μL of each sample was added to 96-well plates, followed by the addition of 200 μL of Bradford reagent. Results were obtained using an ELISA microplate reader (BioTek, Winooski, VT, USA) at 595 nm and expressed as µg/mL [52,53].

### 4.11. Quantitative Determination of Myeloperoxidase Levels

The myeloperoxidase levels were determined in the carrageenan-induced paw edema and in the zymozan-induced air-pouch models. At the end of the carrageenan-induced paw edema experiment, the animals were euthanized, and their right hind paws were removed to determine the myeloperoxidase (MPO) levels, as previously described in the literature, with few modifications [54]. The paw tissues were dispersed in 0.5% hexadecyltrimethylammonium bromide buffer (1 mL of buffer for each 50 mg of tissue), sonicated in ice bath for 3 min, and submitted to three cycles of freeze–thawing, followed by 3 min of sonication for MPO extraction. Next, 20 μL of the supernatant, obtained after centrifugation at 10,000 *g* for 10 min at 4 °C, were mixed with 50 mM of potassium phosphate pH 6.0 containing 0.0005% of hydrogen peroxide and 0.167 mg/mL of *O*-dianisidine. The MPO activity was colorimetrically determined using a microplate reader (Epoch-Biotek, Winooski, VT, USA) at 460 nm. One unit of MPO activity was defined as the amount of enzyme that degrades 1 μmol of hydrogen peroxide. The degradation of 1 μmol of hydrogen peroxide was detected as a change in 1.13 × 10^−2^ in the absorbance per minute. The results were expressed as units of MPO activity per gram of paw tissue (UMPO/mg). 

The MPO level in the mice pouch was determined in the exudate (100 μL) by dispersing it in hexadecyltrimethylammonium bromide buffer (HTAB, 0.5%), pH = 6.0, at the ratio of 1:20 (*w/v*). The samples were then sonicated (Sonic-Tech, São Paulo, Brazil) and subjected to three freeze–thaw cycles. The homogenate was centrifuged, and the obtained supernatant was used for the MPO assay. The reaction of MPO with the chromogenic reagent (*O*-dianisidine, phosphate buffer and hydrogen peroxide) results in the formation of a chromophore that has a maximum absorbance at 450 nm. The MPO activity was determined by interpolation in a standard curve, constructed by analyzing the MPO activity of human neutrophils. Considering that an MPO unit (*U*) degrades 1 nmol min^−1^ of hydrogen peroxide at 25 °C, the results were expressed as U/g of tissue. All reagents were purchased from (Sigma-Aldrich).

### 4.12. Determination of Cytokines Concentration

Exudates from the animals’ air pouch were used to measure cytokines levels (IL-1β and TNF-α), using commercial Enzyme-Linked Immunosorbent Assay kits ELISA (R&D Systems, Minneapolis, MN, USA), according to the manufacturer’s protocol. Results were obtained using an ELISA microplate reader (Epoch, BioTek^®^, Winooski, VT, USA) at 450 nm and expressed in ng/mL.

### 4.13. Statistical Analysis

The data were expressed as mean ± standard deviation. Statistical analyses were carried out by one-way analysis of variance (ANOVA) with Tukey’s test, using GraphPad Prism version 5.00 (San Diego, CA, USA). A difference in the mean values of *** or ^###^ *p* < 0.001, ** or ^##^ *p <* 0.01, and * or ^#^ *p <* 0.05 were considered as statistically significant. For the cytotoxicity assays, the data was expressed as mean ± standard deviation of three measurements (*n* = 3). One-way analysis of variance was performed for data analysis using SigmaPlot^®^ (Systat software, San Jose, CA, USA) and Student-Newman-Keuls for mean comparison, to determine if the results were statistically significant.

## 5. Conclusions

The results indicated that the hydroethanolic extract as well as the EtOAc and BuOH fractions of *T. catharinensis* leaves do have anti-inflammatory activity, corroborating the traditional use of this plant in treating inflammatory disorders. Such activity seems to be attributed, in part, to the glycosylated flavonoids identified in the hydroethanolic extract and in the fractions of *T. catharinensis*. Further studies, taking into consideration the complexity of the extract and the inflammatory process, are essential in order to draw clearer conclusions regarding the anti-inflammatory activity of this plant. 

## Figures and Tables

**Table 1:** Flavonoids identified by HPLC–HRESI-MS in the hydroethanolic extract of *T. catharinensis* leaves.

Peak	(*T_R_*) Retention Time (min)	(MS) Mass Spectrometry (M/Z)	MS^2^	UV Max (nm)	Compounds
5	13.5	[M − H]—739.17	739.17–575.09; 500.15; 473.04; 393.02; 338.92; 284.92; 256.94		kaempferol-3-robinobioside-7-rhamnoside
6	13.8	[M − H]—609.07	609.07–564.41; 488.97; 462.96; 408.87; 344.06; 300.95; 270.97; 216.99	211, 264	quercetin-*O*-rutinoside
7	14.5	[M – H]—769.12	769.12–623.13; 477.09; 357.00; 314.98	211, 351	7-methylquercetin-3-galactoside-6″-rhamnoside-3′′′-rhamnoside
8	15.0	[M – H]—769.12	769.12–754.11; 701.12; 638.06; 623.12; 605.10; 579.02; 503.00; 400.05; 356.95; 314.94	211, 351	isorhamnetin-3-galactoside-6″-rhamnoside-3′′′-rhamnoside
12	16.8	[M – H]—593.06	593.06–326.98; 298.96; 284.91	211, 253	kaempferol-*O*-rutinoside
13	16.8	[M – H]—623.05	623.05–595.09; 477.02; 411.93; 356.95; 327.96; 314.95; 299.89; 270.87		isorhamnetin-3-hexoside-6″-rhamnoside
14	19.4	[M – H]—477.00	477.00–357; 326.95; 284.95; 254.93	249	isorhamnetin-3-*O*-glicoside
15	20.1	[M − H]—477.00	477.00–449.05; 356.95; 315.00	210, 253	isorhamnetin-3-*O-*glicoside
16	20.5	[M − H]—477.00	477.00–356.96; 314.92	211, 251	isorhamnetin-3-*O*-glicoside
12	22.3	[M − H]—563.18	563.18–360.71		isoschaftoside

**Table 2:** Anti-inflammatory activity of the hydroethanolic extract and the ethyl acetate (EtOAc) and *n*-butanol (BuOH) fractions of *T. catharinensis* leaves in the zymosan-induced air pouch model.

Groups	Dose (mg/kg)	Cell Migration (×10^6^/mL)	Inhibition (%)
Saline	-	31.00 ± 4.000	-
Dexamethasone	2	12.80 ± 1.300 ***	59
Hydroethanolic extract	50	28.40 ± 1.965	9
Hydroethanolic extract	100	11.25 ± 2.780 ***	64
Hydroethanolic extract	150	7.125 ± 2.461 ***	77
EtOAc fraction	50	7.400 ± 2.040 ***	76
BuOH fraction	50	17.75 ± 2.602 **	43

Values expressed as mean ± standard deviation (S.D.), *n* = 5, *** *p* < 0.001, and ** *p* < 0.01, tested group compared to the saline-treated group.
